# Reporting of anaesthesia and pain management in preclinical large animal models of articular cartilage repair - A long way to go

**DOI:** 10.1016/j.ocarto.2022.100261

**Published:** 2022-04-17

**Authors:** Maria C. Fugazzola, Kimberley E. Wever, Chris van de Lest, Janny de Grauw, Daniela Salvatori

**Affiliations:** a Department of Equine Sciences, Veterinary Medicine, Utrecht University, Utrecht, the Netherlands; b Radboud Medical Center, Nijmegen, the Netherlands; c Department of Biomolecular Health Sciences, Faculty of Veterinary Medicine, Utrecht University, the Netherlands; d Department of Clinical Sciences, Anatomy and Physiology, Faculty of Veterinary Medicine, Utrecht University, Utrecht, the Netherlands

**Keywords:** Cartilage repair, Large animal models, Pain management, Reporting quality, ARRIVE guidelines

## Abstract

Animal models continue to be used to investigate cartilage repair strategies. Adequate anaesthesia and pain management are essential in order to guarantee acceptable animal welfare as well as reproducible experimental results. This systematic review evaluates reporting of anaesthesia and pain management in surgical large animal models (horse, pig, dog, goat and sheep) of (osteo)chondral repair. Manuscripts published between 2015 and 2020 were included after a comprehensive search strategy. Data were evaluated using descriptive statistics and qualitative review. Out of 223 eligible studies, 220 studies contained incomplete information on anaesthetic and pain management. Pre-, intra- and post-operative analgesia were not mentioned in 68%, 94%, and 64% of manuscripts respectively. A total of 176 studies reported that animals underwent general anaesthesia during surgery. Surprisingly, 30% of these articles did not provide any detail on anaesthetic management, while 37% reported using inhalant, hypnotic or sedative drugs only, without mention of analgesics. Pain monitoring was not reported in 87% of manuscripts. The vast majority of preclinical large animal studies on cartilage repair did not meet veterinary clinical standards for anaesthesia and analgesia, and failed to report according to the ARRIVE international guidelines. In light of serious welfare, ethical and translational validity concerns, improvement is urgently needed.

## 1 Introduction

Despite growing societal, ethical and welfare concerns, animal experimentation remains central to the investigation of new therapies for human medicine. Between 2005 and 2015, the number of animals involved in experimental procedures increased globally by almost 40% [1], while between 2015 and 2017, a slight decrease was noted in the EU [2]. Despite progress in computational models and other alternatives to animal testing such as bioreactors or ‘organs on chips’ [[3], [4], [5]], human clinical application of novel therapeutics still requires previous live animal experimentation. For interventions aimed at improving articular cartilage repair, legal frameworks stipulate the need for preclinical investigation in appropriate large animal models [6,7]. This is because the complexity of the in vivo physiology of the mammalian synovial joint cannot yet be reproduced in vitro. Comparison of cartilage and subchondral bone thickness, biomechanics and healing proprieties of articular cartilage between species has led to the conclusion that the goat, sheep, swine, dog and horse are the most appropriate model species for this type of research, because their osteochondral characteristics most resemble those of humans [6,[8], [9], [10]].

Adequacy of anaesthesia and pain management in large animal models for osteochondral repair can have an impact on validity of experimental results, as pain affects limb loading and the healing process of tissues [[11], [12], [13]]. Also, pain management protocols affect reproducibility, extrapolation and translation of experimental results from animal models to human patients [14]. The ARRIVE (Animal Research: Reporting In Vivo Experiments) guidelines are a checklist of information to include in a manuscript to ensure that publications contain enough information to add to the knowledge base. They were first released in 2010 by the UK National Centre for the 3Rs (NC3Rs) and they were recently further revised in order to facilitate reporting and transparency of animal studies. The guidelines have received widespread endorsement from the scientific community and are currently recommended by over a thousand journals [15]. After their introduction [16], systematic reviews critically assessing reporting quality of anaesthesia and pain management in experimental procedures using small laboratory animals have been published [12,14,17]. Similar systematic assessment of standards for experimental research in large animal species seems to lag behind, possibly due to their minor numeric weight within global animal experimentation [1,16,19]. Specifically, evaluation of adequacy of anaesthesia and pain management in large animal experimental models commonly used in studies of (osteo)chondral repair is lacking.

This systematic review targets current practice of anaesthesia and pain management as well as quality of reporting thereof in studies on large animal models (horses, pigs, dogs, goats and sheep) for surgical repair of (osteo)chondral lesions. The first objective is to assess whether in the 5 years following the release of the ARRIVE 2.0 guidelines [16], peer-reviewed scientific manuscripts report the protocols of anaesthetic and analgesic management according to those guidelines. The second objective is to assess whether anaesthesia and pain management agrees with current standards of veterinary clinical care for control of musculoskeletal pain associated with surgical creation of (osteo)chondral defects.

## 2 Material and methods

This systematic review was preregistered on PROSPERO on March 11th^,^ 2021 and is reported according to PRISMA guidelines [20]. Amendments to the review protocol are published on PROSPERO.

### 2.1 Comprehensive search strategy

To identify experimental studies on the creation and repair of (osteo)chondral lesions in horses, pigs, dogs, goats or sheep, Pubmed and Embase (via Ovid) were systematically searched for records published between the January 6, 2015 and the December 31, 2020 without language restrictions (Table S1) The reference lists of included studies, and those of relevant reviews retrieved by our search, were screened by hand for additional eligible studies.

### 2.2 Eligibility criteria

Studies were included when they met the following inclusion criteria: 1) the study described an in vivo experiment in horses, pigs, dogs, goats or sheep; 2) the study described experimentally induced (osteo)chondral lesions in any form, with or without surgical repair methods; 3) the publication was an original full length research article presenting unique data; 4) the manuscript was published between the year 2015 and 2020.

### 2.3 Study selection

After duplicate removal, unique records were screened based on title and abstract by two independent, blinded authors (MF, KW or DS) using Rayyan software [21]. The following exclusion criteria were applied: 1) not an original full research article, 2) not an in vivo animal study, 3) not on species of interest, 4) no surgical (osteo)chondral lesion induced,. Discrepancies were resolved through discussion until consensus was reached or by a third reviewer serving as arbiter. References eligible for inclusion were subsequently retrieved in full and screened based on the full-text using the same exclusion criteria (MF and JG). Discrepancies were resolved by independent screening by a third blinded reviewer (KW). References were excluded if the full-text could not be retrieved. Three articles published in Chinese and one in Russian were translated using Google translate and the help of a native speaker where needed.

### 2.4 Data extraction and synthesis

The following data were extracted: author, year of publication, species, continent where experiment was carried out, experimental lesion site (joint), experimental lesion type (osteochondral/chondral), reporting of anaesthetic protocol (Y/N) including drug type, reporting of respectively preoperative, intraoperative and postoperative analgesia (Y/N) including drug type, and (if postoperative analgesia was provided), duration of treatment and presence of post-operative pain monitoring.

The data extraction was carried out by one author (MF), while a second author (JG) extracted a random sample of 10% of the data. Discrepancies were resolved by discussion, or, if consensus could not be reached, a third reviewer (KW) served as arbiter. Consensus was >90%, so we proceeded with extraction by a single author, according to protocol.

Data on anaesthetic and analgesic drug use, including descriptive statistics on frequency of reporting (Y versus N), the specific treatment regimens used, and the adherence to item 9 of the ARRIVE 2.0 guidelines) are reported in a narrative synthesis (see https://arriveguidelines.org/sites/arrive/files/documents/ARRIVE%20guidelines%202.0%20-%20English.pdf- last reference access March 30, 2022, and Supplementary file 3 for the description of surgical procedures in item 9). Correlation analysis between year of publication and analgesia and anaesthesia reporting was performed using Spearman's non-parametric correlation test.

## 3 Results

### 3.1 Study selection process

**Fig. 1:**
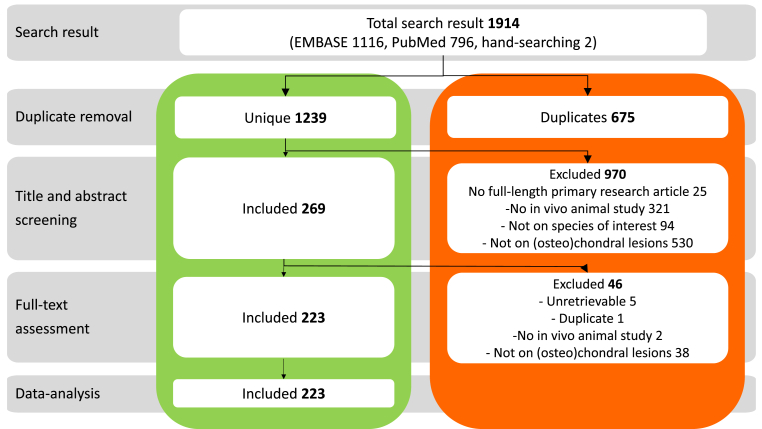
Flowchart of manuscript search and selection process. The right hand panel shows number of excluded manuscripts at each stage, with the reason for exclusion.

The search string yielded 1914 references. After removal of duplicates, 1239 references were available for screening, which resulted in 269 studies that described experimental repair of cartilage defects in in vivo large animal models. Of these 269 studies, 38 were excluded during full-text assessment because they did not involve (osteo)chondral lesions. A further five studies were excluded because the full text manuscripts could not be retrieved, one was a duplicate record, and two were not in-vivo studies. Finally, 223 papers met the inclusion criteria for this review (Fig. 1). A detailed characteristics table of all included studies can be found in the supplementary material (S2a, S2b).

### 3.2 Overview of study characteristics

**Fig. 2:**
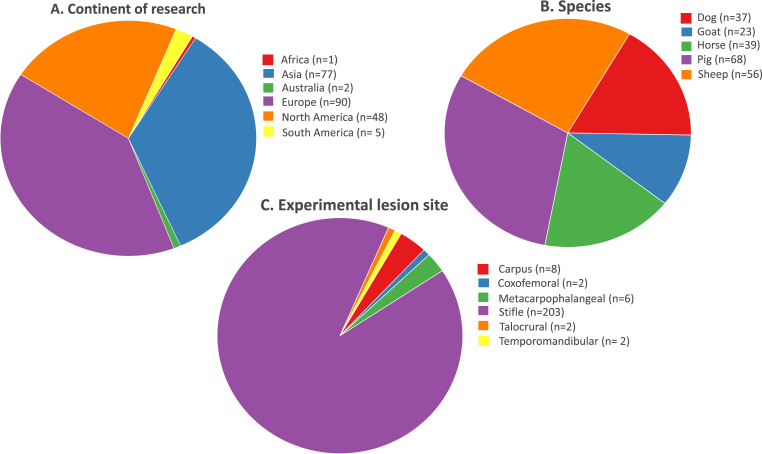
Graphical representation of the general characteristics of the included studies. A. Continent where animal experiment was performed; B. Large animal model species used; C. Anatomical joint where (osteo)chondral experimental lesion was created.

Pigs and sheep were the most frequently used species, comprising 30% (68/223) and 25% (56/223) of the total number of studies respectively, followed by horse (17%, 39/223), dog (16%, 37/223) and goat (10%, 23/223), respectively. Most studies were conducted in Europe (40%), Asia (34%) and North America (22%). The most common anatomical site for experimental lesion creation was the stifle joint (91%, 203/223) (Fig. 2). In 176 of 223 studies (79%), the created lesions were osteochondral, while in 47/273 (21%) studies, lesions were chondral and so did not penetrate the subchondral bone plate.

#### 3.2.1 Pain monitoring and compliance with ARRIVE 2.0 guidelines

Pain monitoring was mentioned in only 13% (28/223) of manuscripts. Overall, reporting of pre-, intra- and post-operative administration of pain medication was present in 2% (4/223) of the articles, while only three studies (1%) were in full compliance with item 9 of the ARRIVE 2.0 guidelines (Fig S3). There was no significant correlation between the year of publication and the reporting of anaesthesia or analgesia (Fig s4).

### 3.3 Reporting and adequacy of anaesthetic management

**Fig. 3:**
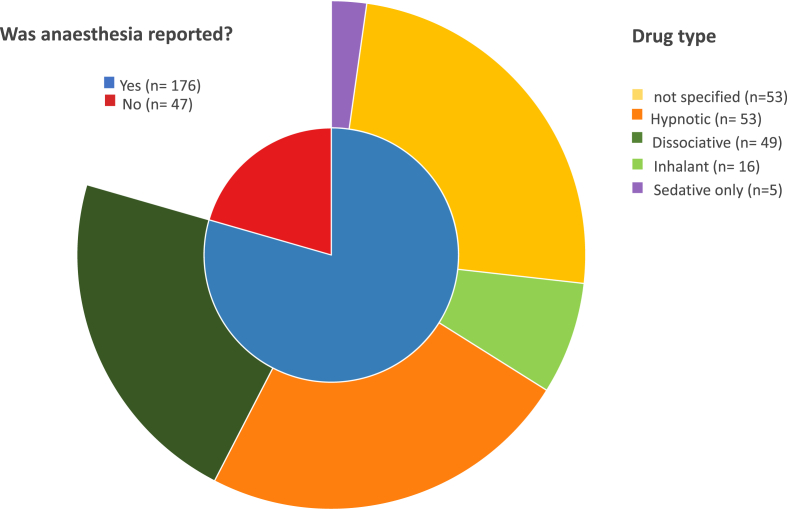
Frequency of reporting general anaesthesia and anaesthetic drugs used in the included studies.

A total of 176 out of 223 studies (79%) mentioned that general anaesthesia was performed; the remaining studies did not. Of the former, 30% (54/176) only mentioned that general anaesthesia was used, without further detail. Of the studies reporting anaesthetic drug use, a hypnotic, dissociative, or inhalant anaesthetic was administered in 30% (53/176), 27% (49/176) and 9% (16/176) respectively, whilst in 3% (5/176) of cases only sedatives were reported (Fig. 3). When a hypnotic, inhalant or sedative anaesthetic were used (lacking intrinsic analgesia), 37% (27/74) of the manuscripts did not report the use of additional pain medication.

### 3.4 Reporting and adequacy of pain management

**Fig. 4:**
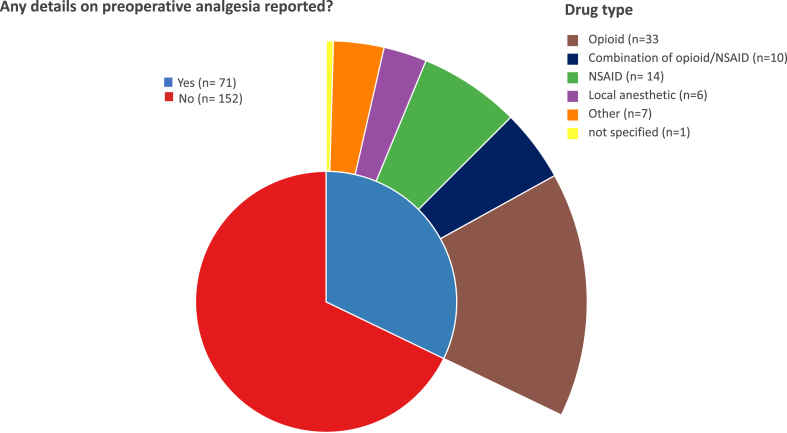
Frequency of reporting preoperative analgesia and analgesic drugs used in the included studies. NSAID: non-steroidal anti-inflammatory drug.

Administration of analgesic drugs before surgery was not reported in 68% of the studies (Fig. 4). Those studies reporting provision of analgesia administered opioids, non-steroidal anti-inflammatory drugs (NSAIDs), or a combination of opioids/NSAIDs/local anaesthetic agent in 46% (33/71), 20% (14/171), 14% (10/171), and 8% (6/171) of cases, respectively. Seven studies reported the use of other analgesic drugs, while one study did not specify the analgesic drug type.

**Fig. 5:**
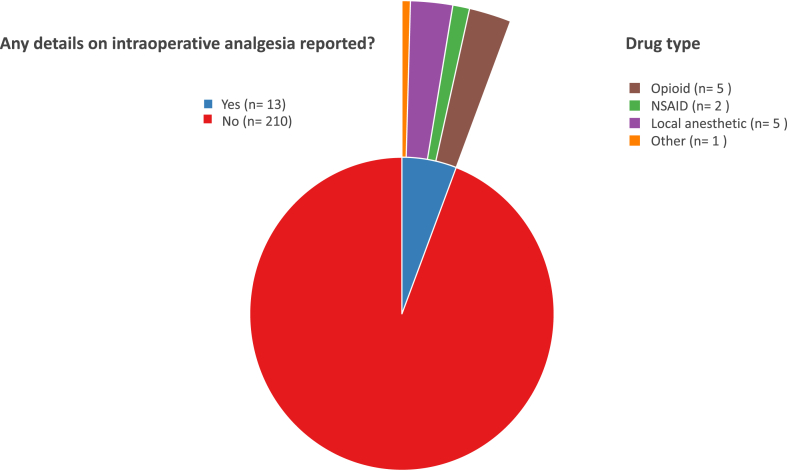
Frequency of reporting intraoperative analgesia and analgesic drugs used in the included studies. NSAID: non-steroidal anti-inflammatory drug.

Intraoperative provision of analgesic drugs was not detailed in 94% (210/223) of manuscripts (Fig. 5). In the 13 studies that did report intra-operative analgesia, opioids and local anaesthetics were administered in 5, while NSAIDs were reported in two.

**Fig. 6:**
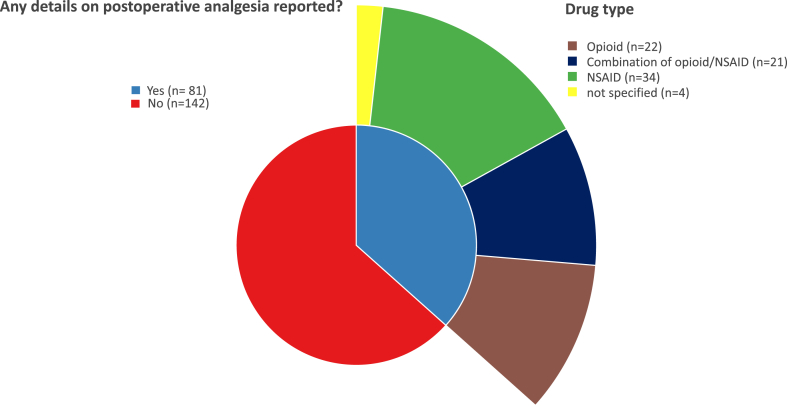
Frequency of reporting postoperative analgesia and analgesic drugs used in the included studies. NSAID: non-steroidal anti-inflammatory drug.

Use of post-operative pain medication was not reported in 64% (142/223) of manuscripts (Fig. 6). Of those that did, 42% (34/81) reported NSAID administration, 27% (22/81) opioids, 26% (21/81) a combination of NSAIDs and opioids, and 5% (4/81) did not specify drug type (Fig. 4, Fig. 5, Fig. 6). Of those studies reporting postoperative analgesia, duration of treatment was not specified in 28% (23/81) of cases. Of the remaining studies, analgesic drugs were provided for up to three days after surgical intervention in 32% (26/81) of the studies, while 39% (32/181) reported provision of analgesia for more than 3 days post-operatively.

## 4 Discussion

The vast majority of preclinical large animal (osteo)chondral defect repair studies included in the present review did not meet the ARRIVE 2.0 guidelines for reporting on in vivo animal experimentation. Also, a large proportion of studies did not meet current veterinary clinical standards for anaesthesia and analgesia provision for orthopaedic surgery. The surgical procedure for (osteo)chondral defect creation is invasive and the associated somatic pain considered moderate to severe. Veterinary literature and practice guidelines support the need for balanced anaesthesia and pain management for such procedures [[22], [23], [24]]. When this type of surgery is performed for the purpose of a preclinical study, the approach to anaesthetic and pain management is particularly crucial, as both pain and the drugs used to manage pain not only affect animal welfare but also the reproducibility and external validity of research outcomes.

### 4.1 Biological and ethical aspects of pain management for (osteo)chondral defect surgeries

In order to create an experimental full-thickness cartilage lesion or an osteochondral lesion, in most cases an arthrotomy is performed in order to gain better visualisation of the articular surface. Mammalian cartilage is aneural, but all joint soft tissues and the subchondral bone are rich in sensory innervation [[25], [26], [27]]. Provision of perioperative analgesia is therefore mandatory for humane animal treatment. Aside from locoregional anaesthetic techniques, combination of a non-steroidal anti-inflammatory drug with an opioid provides the most reliable peri-operative analgesia for painful surgical procedures in mammalian species [28]. Our review showed that only 10 out of 84 studies that reported analgesic drug administration described pre-operative use of a combination of opioids and NSAIDs, while post-operatively this was reported in only 25% of these studies.

Provision of analgesic drugs is required whenever anaesthetic agents are used that do not provide intrinsic anti-nociceptive effects, such as inhalants or hypnotic agents. In human clinical patients, in addition to pre-operative analgesic administration, extra medication is provided intraoperatively, during anaesthesia, when the planned intervention is potentially painful [29]. In our review, when inhalant or hypnotic anaesthetics were given, 37% of studies did not report the use of analgesics. This means that the animal may have been unconscious, but subcortical nociceptive pathways during surgery were active. The possible consequence is the activation of a so-called pain-memory, leading to worse postoperative pain severity and duration [30]. Our results showed that 94% of the studies did not report any intra-operative analgesia provision, which in the authors’ opinion has clear ethical implications.

Aside from its obvious impact on animal welfare, un (der)treated pain has been shown to influence wound healing, which is critical for recovery after surgical interventions. Pain, and indirectly stress caused by pain, can significantly delay wound healing in mice and humans [11,13]. Also, pain can induce altered weight bearing patterns, which has been shown to influence the healing of (subchondral) bone [[31], [32], [33]]. This is highly pertinent in the context of repair of osteochondral tissues, as joint biomechanics and the extent of weightbearing due to pain may influence the healing process and thereby the experimental outcome. On the other hand, results from experimental large animal and human studies have been inconclusive on whether the use of analgesics like NSAIDS could impair fracture healing and delay union [34,35]. Still, by not reporting or omitting the use of analgesics in experimental settings, its effect in clinical use could show different, not-reproducible outcome which again, underscores the need for proper reporting. The absence of pain monitoring in 87% of the reviewed manuscripts is worrisome, as this suggests that not only may insufficient analgesia have gone unnoticed (potentially affecting tissue response and study outcome), but any side effects of analgesics were likely not addressed either. Effective analgesia provision and detection of adverse effects requires comprehensive clinical assessment including regular pain monitoring.

### 4.2 Effects of non-standardized reporting and the ARRIVE 2.0 guidelines

Standardization of principles and introduction of guidelines for reporting preclinical research are a major tool for improving reproducibility of experimental results [36]. Incomplete reporting of experimental studies is associated with risk of bias in in vivo veterinary research [37]. Worryingly, details on experimental procedures in animal studies continue to be underreported in general [12]. A review focussing on anaesthetic use and monitoring in laboratory animal studies that were published in high impact-factor journals pointed out that the quality of reporting remained low even after the first introduction of the ARRIVE guidelines [38].

While the ARRIVE checklist is only one among several emerging guidelines intended to improve reporting of biomedical research methods, they have been formally endorsed by over 300 scientific journals and are included in the US National Research Council Institute for Laboratory Animal Research indications for reporting experimental animal research [15]. The ARRIVE guidelines include 10 essential reporting items, of which item number 9 provides specific details on how to describe an experimental procedure conducted on a live animal. Furthermore, if the experiment entails a surgical procedure, the guideline stipulates ten additional points to be addressed [16].

The current review did not aim to verify overall ARRIVE guideline adherence, nor the extent to which studies met all stipulated items for reporting of surgical procedures; rather, those items most pertinent to anaesthetic and analgesic management were selected. We specifically assessed reporting of the anaesthetic(s) used; pre-, intra-, and post-operative analgesic regimen; and presence of pain monitoring through observation of behaviour or measurement of physiological variables. Discomfortingly, even when limiting our assessment to only these three basic elements of anaesthetic and pain management, 98% of studies failed to report all three core items. The journals in which the only three compliant manuscripts were published officially endorse the ARRIVE guidelines; importantly however, none of the other manuscripts included in this review that were published in the same journals met these minimum reporting requirements. Our results for cartilage repair studies in large animals are in line with other reviews on laboratory animal species (e.g. rabbits and rodents), showing that compliance levels and the impact of reporting guidelines like ARRIVE on improving study reproducibility is still very limited [17,19,39,40]. Furthermore, the fact that no improvement in reporting quality could be shown over the five years since the second introduction of the guidelines is disappointing.

## 5 Conclusions

Out of 223 recently published manuscripts on large animal models of articular cartilage defect repair, only three covered the basic anaesthesia and analgesia reporting items for surgical procedures outlined in the ARRIVE 2.0 guidelines, despite widespread formal endorsement of these guidelines by journals in this field. Here, editors and peer reviewers have a crucial role in improving standards of performance and reporting of animal research. Importantly, the lack of information on anaesthesia, pain management and pain monitoring in large animal surgical models for (osteo)chondral repair may lead to unreliable and irreproducible results, undermining the basic premise underlying preclinical animal studies: translatability of outcomes to the human clinical situation. The eventual failure to produce translational results carries the ethical burden of unnecessary animal use as well as great economic loss [36,40].

## Author contributions

MF performed the systematic research, the screening and data extraction and wrote the manuscript. KW contributed with the methods and protocols for the review, performed part of the screening process and critically reviewed the manuscript. CvL helped with the data extraction and analysis. JdG conceived the study, performed part of the screening process and critically reviewed the manuscript. DS conceived the study and critically reviewed the manuscript.

## Funding

ZonMW grant number 114024152.

## Declaration of competing interest

The authors have no competing interest to declare.
