# Supplementary material for: Reporting of anaesthesia and pain management in preclinical large animal models of articular cartilage repair - A long way to go
Source: Osteoarthr Cartil Open. 2022 Apr 17;4(2):100261. doi: 10.1016/j.ocarto.2022.100261 (PMC9718186; doi:10.1016/j.ocarto.2022.100261)

***^Supplementary information^***

Supplementary file Table 1– full search strings for PubMed and EMBASE

| *PubMed* | 06012021 | 796 hits |
| --- | --- | --- |
| #1 | (subchondral arthroplasty)  **OR** (cartilage **AND** damage) | ("Arthroplasty"[Mesh:NoExp] OR "Arthroplasty, Subchondral"[Mesh] OR "Fractures, Cartilage"[Mesh] OR "subchondral drill*"[tiab] OR “arthroscopic drill*”[tiab] OR “subchondral arthropl*”[tiab] OR "subchondral abrasion"[tiab] OR "subchondral abrasions"[tiab]) **OR** ("Cartilage, Articular"[Mesh] OR "Chondrogenesis"[Mesh] OR cartilage[tiab] OR chondral[tiab] OR chondrogenic[tiab] OR chondrogenesis[tiab])  **AND** (defect[tiab] OR defects[tiab] OR repair[tiab] OR implant[tiab] OR implants[tiab] OR microfrac*[tiab] OR operat*[tiab] OR surgery[tiab] OR surgeries[tiab] OR transplant[tiab] OR transplants[tiab] OR transplantation[tiab] OR transplantations[tiab]) |
| #2 | Animal models | Dogs[MeSH] OR Horses[MeSH] OR Sheep, Domestic[MeSH] OR Swine[MeSH] OR Goats[MeSH] OR dog[tiab] OR dogs[tiab] OR canis[tiab] OR canine[tiab] OR horse[tiab] OR horses[tiab] OR equus[tiab] OR equine[tiab] OR pony[tiab] OR ponies[tiab] OR foal[tiab] OR foals[tiab] OR sheep[tiab] OR ovis[tiab] OR ovine[tiab] OR mouflon[tiab] OR mouflons[tiab] OR swine[tiab] OR suidae[tiab] OR pig[tiab] OR pigs[tiab] OR piglet[tiab] OR piglets[tiab] OR hog[tiab] OR hogs[tiab] OR warthog[tiab] OR warthogs[tiab] OR phacochoerus[tiab] OR porcine[tiab] OR goat[tiab] OR goats[tiab] OR capra [tiab] OR capras [tiab] OR caprine[tiab] |
| #3 | Cartilage damage in animal models | #1 AND #2 |
| #4 | Limit pub date | #3 AND "2015/01/01"[Date - Publication] : "2020/12/31"[Date - Publication] |
| #5 | Remove reviews | #4 NOT review[ptyp] |
| *#6* | *Retrieve reviews for separate screening (29 hits)* | *#5 NOT #6* |
| *EMBASE* | 06012021 | 1116 hits |
| #1 | (subchondral arthroplasty)  **OR** (cartilage **AND** damage) | arthroplasty/ OR exp cartilage transplantation/ or exp cartilage injury/ or exp cartilage graft/ or exp cartilage degeneration/ or exp cartilage fracture/ OR (subchondral drill* OR arthroscopic drill* OR subchondral arthropl* OR subchondral abrasion OR subchondral abrasions).ti,ab.) **OR** ((articular cartilage/ OR exp cartilage/ OR chondrogenesis/ OR (cartilage OR chondral OR chondrogenic OR chondrogenesis).ti,ab.) **AND** (defect OR defects OR repair OR implant OR implants OR microfrac* OR operat* OR surgery OR surgeries OR transplant OR transplants OR transplantation OR transplantations).ti,ab.) |
| #2 | Animal models | exp dog/ OR exp horse/ OR exp sheep/ OR exp pig/ OR exp warthog/ OR exp goat/ OR (dog OR dogs OR canis OR canine OR horse OR horses OR equus OR equine OR pony OR ponies OR foal OR foals OR sheep OR ovis OR ovine OR mouflon OR mouflons OR swine OR suidae OR pig OR pigs OR piglet OR piglets OR hog OR hogs OR warthog OR warthogs OR phacochoerus OR porcine OR goat OR goats OR capra OR capras OR caprine).ti,ab. |
| #3 | Cartilage damage in animal models | 1 AND 2 |
| #4 | Limit pub date | Limit 3 to yr=”2015 – 2020” |
| #5 | Remove reviews etc. | 4 AND (article OR article in press OR conference paper).pt. |
| *#6* | *Retrieve reviews for separate screening (49 hits)* | *4 AND review.pt.* |

Supplementary file 2a: Bibliographical details and animal model characteristics of the included studies.

| **StudyID** | **Journal** | **Continent** | **Species** | **Lesion type** | **Lesion**  **site** |
| --- | --- | --- | --- | --- | --- |
| Airapetov2019 | J Orthop | Asia | Sheep | OC | stifle |
| Airapetov2019 | Sovrem Tekhnologii Med | Asia | Sheep | OC | stifle |
| Aisenbrey2019 | Biochem Biophys Res Commun | N-America | Pig | OC | stifle |
| Ao2019 | Am J Sports Med | Asia | Pig | C | stifle |
| Asen2018 | FASEB Journal | Europe | Pig | OC | stifle |
| Baba2018 | Am J Sports Med | Asia | Dog | OC | stifle |
| Beck2016 | Am J Sports Med | Australia | Sheep | OC | stifle |
| Bell2017 | Cartilage | N-America | Sheep | OC | stifle |
| Bertoni2020 | PLoS One | Europe | Horse | OC | MCP |
| Bonadio2017 | J Exp Orthop | Europe | Pig | C | stifle |
| Bornes2018 | Tissue Eng Part A | N-America | Sheep | C | stifle |
| Bothe2019 | Int J Mol Sci | Europe | Pig | C | stifle |
| Boyer2020 | Front Bioeng Biotechnol | Europe | Dog | OC | stifle |
| Bozkurt2019 | J Orthop Surg Res | Europe | Sheep | OC | stifle |
| Brimmo2018 | J Orthop Res | N-America | Dog | OC | stifle |
| Broeckx2019 | Am J Vet Res | Europe | Horse | OC | MCP |
| Broeckx2019 | Equine Vet J | Europe | Horse | OC | MCP |
| Caminal2016 | Cytotechnology | Europe | Sheep | OC | stifle |
| Cernohorsky2015 | Acta orthopaedica | Europe | Goat | OC | talocrural |
| Chen2020 | J Tissue Eng Regen Med | Asia | Pig | OC | stifle |
| Chen2019 | Knee Surg Sports Traumatol Arthrosc | Asia | Pig | OC | stifle |
| Choi2016 | J Orthop Res | Asia | Dog | OC | stifle |
| Christensen2017 | J Exp Orthop | Europe | Pig | OC | stifle |
| Christensen2015 | Am J Sports Med | Europe | Pig | C | stifle |
| Christensen2016 | Am J Sports Med | Europe | Pig | OC | stifle |
| Chu2018 | J Bone Joint Surg Am | N-America | Horse | OC | stifle |
| Cokelaere2020 | Pferdeheilkunde | Europe | Horse | OC | stifle |
| Cole2015 | Orthop J Sports Med | N-America | Horse | C | stifle |
| Cook2016 | Am J Sports Med | N-America | Dog | OC | stifle |
| Crist2016 | J Orthop Translat | N-America | Dog | OC | stifle |
| Critchley2020 | Acta Biomaterialia | Europe | Goat | OC | stifle |
| Crovace2019 | Vet Sci | Europe | Sheep | OC | stifle |
| Cucchiarini2018 | Am J Sports Med | Europe | Pig | OC | stifle |
| de Girolamo2015 | Regen Med | Europe | Pig | OC | stifle |
| Delco2020 | Am J Sports Med | N-America | Horse | OC | talocrural |
| Di Bella2018 | J Tissue Eng Regen Med | Australia | Sheep | C | stifle |
| Dickinson2017 | Stem Cells | Europe | Sheep | C | stifle |
| Dominguez Perez2019 | Knee Surg Sports Traumatol Arthrosc | Europe | Sheep | C | stifle |
| Eldridge2020 | Sci Transl Med | Europe | Sheep | OC | stifle |
| Fan2016 | J Biomater Tissue Eng | Asia | Dog | OC | stifle |
| Favreau2020 | Nanomedicine | Europe | Sheep | OC | stifle |
| Feeney2019 | J Orthop Res | N-America | Horse | OC | MCP |
| Fernandes2018 | Stem Cell Rev Rep | S- America | Pig | C | stifle |
| Fisher2016 | Cartilage | N-America | Pig | OC | stifle |
| Fisher2015 | Tissue Eng Part A | N-America | Pig | OC | stifle |
| Fortier2016 | Am J Sports Med | N-America | Horse | C | stifle |
| Franklin2018 | J Knee Surg | N-America | Dog | OC | stifle |
| Franklin2020 | J Orthop Res | N-America | Dog | OC | stifle |
| Friedman2018 | Cartilage | N-America | Pig | OC | stifle |
| Frisbie2015 | J Bone Joint Surg Am | N-America | Horse | C | stifle |
| Fujie2015 | J Biomech | Asia | Pig | C | stifle |
| Gao2017 | Sci Rep | Europe | Pig | OC | stifle |
| Garcia2015 | Curr Stem Cell Res Ther | Europe | Sheep | OC | stifle |
| Gelse2015 | J Orthop Res | Europe | Sheep | C | stifle |
| Geraghty2015 | J Orthop Surg Res | Europe | Goat | OC | stifle |
| Giretova2019 | Appl Biochem Biotechnol | Europe | Sheep | C | stifle |
| Goebel2017 | Osteoarthritis Cartilage | Europe | Sheep | C | stifle |
| Goodrich2016 | J Bone Joint Surg Am | N-America | Horse | OC | stifle |
| Griffin2015 | J Biomech | N-America | Horse | C | stifle |
| Griffin2016 | J Orthop Res | N-America | Horse | OC | stifle |
| Gullberg2019 | Folia Veterinaria | Europe | Sheep | OC | stifle |
| Gurer2018 | J Orthop Surg Res | Europe | Sheep | OC | stifle |
| H2015 | Open Orthop J | Europe | Sheep | OC | stifle |
| Ha2015 | Stem Cells Transl Med | Asia | Pig | OC | stifle |
| He2017 | Sci Rep | Asia | Pig | OC | stifle |
| Henson2020 | J Orthop Res | Europe | Sheep | OC | stifle |
| Higgins2018 | Hand Surg | Europe | Pig | OC | stifle |
| Hindle2016 | Stem Cells Dev | Europe | Sheep | C | stifle |
| Hopper2015 | PLoS One | Asia | Pig | OC | stifle |
| Hoshi2016 | Int J Oral Maxillofac Surg | Asia | Dog | OC | stifle |
| Hou2019 | Chinese J Tissue Engineering Res | Asia | Goat | OC | stifle |
| Howard2015 | J Orthop Res | Europe | Sheep | C | stifle |
| Hsieh2018 | Int J Mol Sci | Asia | Pig | OC | stifle |
| Husby2016 | Vet Surg | N-America | Horse | OC | stifle |
| Jakobsen2017 | BMC Musculoskelet Disord | Europe | Sheep | OC | stifle |
| Jia2018 | ACS Appl Mater Interfaces | Asia | Goat | OC | stifle |
| Jin2020 | Chinese J Tissue Engineering Res | Asia | Dog | OC | stifle |
| Kazemi2017 | Bone & joint research | Asia | Dog | OC | stifle |
| Kazemi2015 | Iran Red Crescent Med J | Asia | Dog | OC | stifle |
| Keller2019 | Nat Commun | Europe | Sheep | OC | stifle |
| Kim2018 | Knee | Asia | Dog | OC | stifle |
| Kim2015 | PLoS One | Asia | Pig | OC | stifle |
| Kim2020 | Tissue Eng Part A | N-America | Pig | OC | stifle |
| Kitamura2016 | J Biomed Mater Res A | Asia | Sheep | OC | stifle |
| Kok2019 | Cartilage | Europe | Goat | OC | stifle |
| Kon2015 | Arthroscopy | N-America | Goat | OC | stifle |
| Kon2020 | J Orthop Surg Res | Asia | Goat | OC | stifle |
| Kondo2019 | Am J Sports Med | Asia | Pig | OC | stifle |
| Korthagen2019 | Vet J | Europe | Horse | OC | stifle |
| Lan2016 | J Biomater Tissue Eng | Asia | Sheep | OC | stifle |
| Levingstone2016 | Biomaterials | Europe | Goat | OC | stifle |
| Li2016 | Exp Ther Med | Asia | Dog | OC | stifle |
| Li2018 | Sci Rep | Asia | Dog | OC | stifle |
| Lin2017 | Bone & joint research | Asia | Pig | OC | stifle |
| Lin2020 | Am J Sports Med | Asia | Pig | OC | stifle |
| Ling2020 | Stem Cell Rep | Asia | Pig | OC | stifle |
| Little2016 | J Exp Orthop | N-America | Dog | C | coxofemoral |
| Lv2015 | Bone & joint research | Asia | Dog | OC | stifle |
| Lydon2019 | Cartilage | Europe | Sheep | OC | stifle |
| Madry2020 | Adv Mater | Europe | Pig | C | stifle |
| Mancini2020 | Biofabrication | Europe | Horse | OC | stifle |
| Mancini2017 | Tissue Eng Part C Methods | Europe | Horse | OC | stifle |
| Maninchedda2015 | PLoS One | Europe | Horse | C | MCP |
| Manunta2016 | Joints | Europe | Sheep | OC | stifle |
| Marteau2015 | Current Tissue Engineering | Europe | Horse | OC | MCP |
| Martinez-Carranza2016 | Cartilage | Europe | Sheep | OC | stifle |
| Martinez-Carranza2019 | Osteoarthritis Cartilage | Europe | Sheep | OC | stifle |
| McCarrel2017 | Cartilage | N-America | Horse | OC | stifle |
| McCarty2016 | Am J Sports Med | N-America | Dog | OC | stifle |
| Meppelink2016 | Tissue Eng Part A | N-America | Pig | OC | stifle |
| Mickevicius2020 | Cartilage | Europe | Goat | OC | stifle |
| Miki2016 | Teikyo Med J | Asia | Dog | C | stifle |
| Mohan2015 | Regen Med | N-America | Sheep | OC | stifle |
| Moller2020 | J Orthop Res | Europe | Horse | C | carpus |
| Moore2018 | Orthopedics | N-America | Pig | OC | stifle |
| Mrosek2016 | Bone & joint research | N-America | Sheep | OC | stifle |
| Muhonen2016 | J Orthop Res | Europe | Pig | C | stifle |
| Mumme2016 | Tissue Eng Part A | Europe | Goat | OC | stifle |
| Murata2018 | J Orthop Surg Res | Asia | Pig | OC | stifle |
| Murata2015 | Tissue Eng Regen Med | Asia | Pig | OC | stifle |
| Nelson2019 | J Orthop Res | Europe | Horse | OC | stifle |
| Nelson2020 | Osteoarthritis Cartilage | N-America | Horse | OC | stifle |
| Nie2020 | Biomaterials | Asia | Pig | C | stifle |
| Niemela2019 | Acta Vet Scand | Europe | Horse | OC | carpus |
| Nixon2017 | J Bone Joint Surg Am | N-America | Horse | OC | stifle |
| Novak2016 | Tissue Eng Part A | N-America | Sheep | OC | stifle |
| Olesen2020 | Arch Orthop Trauma Surg | Europe | Pig | C | stifle |
| Olesen2019 | Cartilage. | Europe | Pig | OC | stifle |
| Olive2020 | Vet Surg | Europe | Sheep | OC | stifle |
| Oliver2020 | J Orthop Res | N-America | Dog | OC | stifle |
| Onodera2020 | Regen Ther | Asia | Dog | OC | stifle |
| Orth2016 | J Clin Med | Europe | Sheep | OC | stifle |
| Orth2020 | Am J Sports Med | Europe | Sheep | OC | stifle |
| Ortved2015 | Mol Ther | N-America | Horse | C | stifle |
| Park2015 | Cell transplantation | Asia | Dog | OC | stifle |
| Passino2017 | Clin Interv Agin | Europe | Sheep | OC | stifle |
| Patel2020 | Cartilage | N-America | Pig | OC | stifle |
| Peal2020 | J Orthop Res | N-America | Horse | OC | stifle |
| Peck2015 | Sci Rep | Asia | Pig | C | stifle |
| Peretti2017 | J Biol Regul Homeost Agents | Europe | Sheep | OC | stifle |
| Perez-Silos2019 | Int J Mol Sci | S-America | Pig | OC | stifle |
| Pfeifer2015 | Orthop J Sports Med | N-America | Pig | OC | stifle |
| Pfeifer2017 | Tissue Eng Part C Methods | N-America | Pig | C | stifle |
| Pilichi2018 | BMC Vet Res | Europe | Sheep | OC | stifle |
| Qiao2020 | Theranostics | Asia | Sheep | C | stifle |
| Reesink2018 | Front Vet Sci | N-America | Horse | OC | carpus |
| Reesink2017 | Osteoarthritis Cartilage | N-America | Horse | OC | carpus |
| Ribitsch2018 | Dis Model Mech | Europe | Sheep | OC | stifle |
| Roth2020 | Knee | Europe | Sheep | OC | stifle |
| Ruvinov2019 | J Orthop Translat | Europe | Pig | OC | stifle |
| Sabry2018 | J Musculoskeletal Res | Africa | Dog | C | stifle |
| Salkeld2016 | J Orthop Surg Res | N-America | Dog | OC | stifle |
| Salonius2019 | Connect Tissue Res | Europe | Horse | OC | carpus |
| Santos2019 | Res Vet Sci | S-America | Horse | C | carpus |
| Sarin2020 | Osteoarthritis Cartilage | Europe | Horse | C | stifle |
| Sarin2018 | Sci Rep | Europe | Horse | C | stifle |
| Schagemann2016 | Cartilage | Europe | Sheep | OC | stifle |
| Schell2019 | Knee Surg Sports Traumatol Arthrosc | Europe | Sheep | OC | stifle |
| Schinhan2020 | Am J Sports Med | Europe | Sheep | C | stifle |
| Schone2016 | Eur Cell Mate | Europe | Sheep | OC | stifle |
| Schwarz2019 | PLoS One | Europe | Pig | C | stifle |
| Seo2015 | Res Vet Sci | Asia | Horse | OC | stifle |
| Sheu2017 | Knee | Asia | Pig | OC | stifle |
| Silva2018 | Acta Scientiae Veterinariae | S-America | Goat | OC | stifle |
| Smith2016 | Vet Radiol Ultrasound | N-America | Horse | OC | carpus |
| Sobol2017 | J Biomed Opt | Asia | Pig | C | coxofemoral |
| Sosio2015 | Tissue Eng Part A | Europe | Pig | OC | stifle |
| Stannard2015 | Orthop J Sports Med | Europe | Dog | OC | stifle |
| Stefani2020 | Acta Biomaterialia | N-America | Dog | OC | stifle |
| Stefani2020 | Biotechnol Bioeng | Europe | Dog | OC | stifle |
| Stewart2019 | J Orthop Res | N-America | Horse | C | stifle |
| Sun2016 | Acta Orthop Traumatol Turc | Asia | Goat | OC | stifle |
| Sun2020 | J Biomed Mater Res A | Asia | Dog | OC | stifle |
| Szivek2017 | J Biomed Mater Res B | N-America | Dog | OC | stifle |
| Tang2019 | Med Sci Monit | Asia | Pig | OC | stifle |
| Theruvath2019 | Radiology | N-America | Pig | C | stifle |
| Tomaszewski2019 | J Orthop Surg Res | Europe | Pig | OC | stifle |
| Toth2015 | Osteoarthritis Cartilage | N-America | Goat | OC | stifle |
| Tothova2019 | Animals (Basel) | Europe | Pig | OC | stifle |
| Tothova2019 | Materials (Basel) | Europe | Sheep | OC | stifle |
| Tuska2016 | Acta veterinaria Hungarica | Europe | Horse | OC | stifle |
| Uklejewski2016 | Materials (Basel) | Europe | Pig | OC | stifle |
| Uto2018 | Regen Ther | Asia | Pig | OC | stifle |
| Vahedi2020 | Cell Tissue Bank | Asia | Sheep | C | stifle |
| Vahedi2019 | Tissue Cell | Asia | Sheep | C | stifle |
| Vikingsson2015 | Int J Artif Organs | Europe | Sheep | OC | stifle |
| Vindas Bolanos2017 | Osteoarthritis Cartilage | Europe | Horse | OC | stifle |
| Wang2019 | J Biomed Mater Res A | Asia | Pig | OC | stifle |
| Wang2017 | J Oral Maxillofac Surg | Asia | Goat | OC | TM |
| Wei2019 | Stem Cell Res Ther | Asia | Goat | OC | stifle |
| Wiegant2015 | Arthritis Rheumatol | Europe | Dog | C | stifle |
| Wiegant2016 | Knee | Europe | Goat | C | stifle |
| Wong2020 | Int J Mol Sci | Asia | Pig | OC | stifle |
| Wu2019 | Artif Cells Nanomed Biotechnol | Asia | Dog | OC | stifle |
| Wu2018 | Artif Cells Nanomed Biotechnol | Asia | Dog | OC | stifle |
| Wu2019 | Tzu Chi Medical Journal | Asia | Pig | OC | stifle |
| Xiao2019 | Orthop Sur | Asia | Pig | OC | stifle |
| Xin2020 | Life (Basel) | Europe | Sheep | OC | stifle |
| Xu2020 | Cartilage. | Asia | Pig | OC | stifle |
| Xu2020 | Am J Sports Med | Asia | Pig | OC | stifle |
| Xue2018 | Biomed Mater | Asia | Pig | OC | stifle |
| Yamasaki2018 | J Equine Sci | Asia | Horse | OC | carpus |
| Yamasaki2019 | J Orthop Res | Asia | Pig | OC | stifle |
| Yamasaki2015 | Regen Ther | Asia | Dog | OC | stifle |
| Yan2019 | Biomed Environ Sci | Asia | Dog | OC | stifle |
| Yan2017 | Regen Biomater | Asia | Pig | OC | stifle |
| Yoon2016 | Tissue Eng Part A | Asia | Dog | OC | stifle |
| Yu2020 | Acta Biomaterialia | Asia | Goat | OC | TM |
| Yucekul2017 | J Tissue Eng | Europe | Sheep | OC | stifle |
| Zedde2016 | Joints | Europe | Sheep | OC | stifle |
| Zedde2017 | Joints | Europe | Sheep | OC | stifle |
| Zhai2018 | Tissue Eng Part A | Asia | Goat | OC | stifle |
| Zhang2020 | Biofabrication | Asia | Goat | OC | stifle |
| Zhang2018 | Biomed Mater | Asia | Goat | OC | stifle |
| Zhang2020 | Front Biosci (Landmark Ed) | Asia | Pig | OC | stifle |
| Zhang2018 | Osteoarthritis Cartilage | Asia | Goat | OC | stifle |
| Zhang2020 | Stem Cell Res Ther | Asia | Goat | C | stifle |
| Zhang2017 | Stem cells Int | Asia | Dog | OC | stifle |
| Zhang2019 | Am J Sports Med | Asia | Pig | C | stifle |
| Zhao2018 | Cells Tissues Organs | Asia | Pig | OC | stifle |
| Zhu2017 | J Biomater Tissue Eng | Asia | Pig | C | stifle |
| Zorzi2015 | Int J Mol Sci | S-America | Sheep | OC | stifle |
| Zuo2016 | J Tissue Eng Regen Med | Asia | Pig | OC | stifle |
| Continent = continent where the research was performed, N = North, S = South, OC = osteochondral, C = chondral, MCP = metacarpophalangeal, TM = temporomandibular | | | | | |

Supplementary file 2b: Reporting of anaesthetic and analgesic use in included studies. NR = Not reported; Hypno = Hypnotic anaesthetic agent; Disso = Dissociative anaesthetic agent; Inhal = Inhalant anaesthetic agent; Local = Local anaesthetic agent; NSAID = Non-steroidal anti-inflammatory drug.

| **StudyID** | **Pain monit** | **Anaes used?** | **Anaes drug class** | **Pre-op analg used?** | **Pre-op analg drug class** | **In-op analg used?** | **In-op analg drug class** | **Post-op analg used?** | **Post-op analg drug class** | **Compliant**  **with**  **ARRIVE**  **Guidelines?** |
| --- | --- | --- | --- | --- | --- | --- | --- | --- | --- | --- |
| Airapetov2019 | NR | NR | NR | NR | NR | NR | NR | NR | NR | No |
| Airapetov2019 | NR | NR | NR | NR | NR | NR | NR | NR | NR | No |
| Aisenbrey2019 | NR | Yes | Inhal | Yes | Opioid | NR | NR | Yes | Opioid | No |
| Ao2019 | NR | Yes | Hypno | Yes | Opioid | NR | NR | NR | NR | No |
| Asen2018 | NR | Yes | Hypno | NR | NR | Yes | NSAID | Yes | Opioid + NSAID + local | No |
| Baba2018 | NR | Yes | Disso | NR | NR | NR | NR | NR | NR | No |
| Beck2016 | NR | Yes | Disso | Yes | Opioid + NSAID + local | NR | NR | Yes | Opioid + NSAID + local | No |
| Bell2017 | Yes | Yes | Disso | Yes | NSAID | NR | NR | Yes | NSAID | No |
| Bertoni2020 | Yes | Yes | Inhal | Yes | NSAID | NR | NR | Yes | NSAID | No |
| Bonadio2017 | Yes | Yes | Disso | NR | NR | NR | NR | Yes | Opioid | No |
| Bornes2018 | Yes | Yes | Disso | NR | NR | NR | NR | Yes | Opioid + NSAID + local | No |
| Bothe2019 | NR | Yes | Disso | NR | NR | NR | NR | Yes | Opioid + NSAID + local | No |
| Boyer2020 | NR | Yes | NR | NR | NR | NR | NR | NR | NR | No |
| Bozkurt2019 | Yes | Yes | NR | NR | NR | NR | NR | NR | NR | No |
| Brimmo2018 | Yes | Yes | NR | NR | NR | NR | NR | Yes | Opioid | No |
| Broeckx2019 | NR | Yes | NR | NR | NR | Yes | Opioid | NR | NR | No |
| Broeckx2019 | NR | NR | NR | NR | NR | Yes | Opioid | NR | NR | No |
| Caminal2016 | Yes | Yes | Hypno | Yes | Opioid | NR | NR | Yes | Opioid + NSAID + local | No |
| Cernohorsky2015 | NR | Yes | NR | NR | NR | NR | NR | NR | NR | No |
| Chen2020 | NR | Yes | Hypno | Yes | Opioid | NR | NR | Yes | Opioid | No |
| Chen2019 | NR | NR | NR | NR | NR | NR | NR | NR | NR | No |
| Choi2016 | NR | Yes | Inhal | Yes | NSAID | NR | NR | Yes | NSAID | No |
| Christensen2017 | NR | Yes | Inhal | Yes | Opioid + NSAID + local | NR | NR | NR | NR | No |
| Christensen2015 | NR | Yes | Hypno | Yes | Local | NR | NR | Yes | NSAID | No |
| Christensen2016 | NR | Yes | Inhal | Yes | Opioid | Yes | Local | NR | NR | No |
| Chu2018 | NR | NR | NR | NR | NR | NR | NR | NR | NR | No |
| Cokelaere2020 | NR | Yes | NR | Yes | NSAID | NR | NR | Yes | NSAID | No |
| Cole2015 | NR | Yes | NR | NR | NR | NR | NR | NR | NR | No |
| Cook2016 | Yes | Yes | Hypno | Yes | Opioid | NR | NR | Yes | Opioid | No |
| Crist2016 | NR | Yes | Hypno | Yes | Opioid | NR | NR | Yes | Opioid | No |
| Critchley2020 | NR | Yes | NR | NR | NR | NR | NR | NR | NR | No |
| Crovace2019 | NR | Yes | Hypno | Yes | NSAID | Yes | Local | Yes | NSAID | Yes |
| Cucchiarini2018 | NR | Yes | Disso | NR | NR | NR | NR | Yes | Opioid + NSAID + local | No |
| de Girolamo2015 | NR | Yes | NR | NR | NR | NR | NR | Yes | NSAID | No |
| Delco2020 | NR | Yes | NR | NR | NR | NR | NR | Yes | NSAID | No |
| Di Bella2018 | NR | Yes | Hypno | Yes | Opioid | NR | NR | Yes | Opioid | No |
| Dickinson2017 | NR | Yes | NR | NR | NR | NR | NR | NR | NR | No |
| Dominguez Perez2019 | NR | Yes | NR | NR | NR | NR | NR | Yes | Opioid | No |
| Eldridge2020 | NR | NR | NR | NR | NR | NR | NR | NR | NR | No |
| Fan2016 | NR | NR | NR | NR | NR | NR | NR | NR | NR | No |
| Favreau2020 | NR | Yes | Disso | NR | NR | NR | NR | Yes | Opioid | No |
| Feeney2019 | NR | NR | NR | NR | NR | NR | NR | NR | NR | No |
| Fernandes2018 | NR | Yes | Hypno | Yes | Opioid | NR | NR | Yes | NSAID | No |
| Fisher2016 | NR | Yes | Inhal | NR | NR | NR | NR | Yes | Opioid + NSAID + local | No |
| Fisher2015 | NR | Yes | Inhal | NR | NR | NR | NR | Yes | Opioid + NSAID + local | No |
| Fortier2016 | NR | Yes | NR | NR | NR | NR | NR | NR | NR | No |
| Franklin2018 | NR | Yes | Hypno | Yes | Opioid | NR | NR | Yes | Opioid + NSAID + local | No |
| Franklin2020 | Yes | Yes | Hypno | Yes | Opioid | NR | NR | Yes | Opioid + NSAID + local | No |
| Friedman2018 | NR | Yes | NR | NR | NR | NR | NR | NR | NR | No |
| Frisbie2015 | NR | Yes | NR | NR | NR | NR | NR | NR | NR | No |
| Fujie2015 | NR | NR | NR | NR | NR | NR | NR | NR | NR | No |
| Gao2017 | NR | Yes | Hypno | Yes | Opioid + NSAID + local | NR | NR | Yes | Opioid | No |
| Garcia2015 | NR | Yes | Disso | NR | NR | NR | NR | NR | NR | No |
| Gelse2015 | NR | Yes | NR | NR | NR | NR | NR | NR | NR | No |
| Geraghty2015 | NR | NR | NR | NR | NR | NR | NR | NR | NR | No |
| Giretova2019 | NR | Yes | Disso | Yes | Opioid | NR | NR | NR | NR | No |
| Goebel2017 | NR | NR | NR | NR | NR | NR | NR | NR | NR | No |
| Goodrich2016 | NR | NR | NR | NR | NR | NR | NR | NR | NR | No |
| Griffin2015 | NR | NR | NR | NR | NR | NR | NR | NR | NR | No |
| Griffin2016 | NR | NR | NR | NR | NR | NR | NR | NR | NR | No |
| Gullberg2019 | NR | Yes | Disso | Yes | Opioid | NR | NR | Yes | NSAID | No |
| Gurer2018 | NR | Yes | Disso | NR | NR | NR | NR | NR | NR | No |
| H2015 | NR | Yes | Sedative | Yes | Other | NR | NR | Yes | NSAID | No |
| Ha2015 | NR | Yes | Disso | NR | NR | NR | NR | Yes | NSAID | No |
| He2017 | NR | NR | NR | NR | NR | NR | NR | NR | NR | No |
| Henson2020 | NR | Yes | NR | NR | NR | NR | NR | NR | NR | No |
| Higgins2018 | NR | Yes | Hypno | NR | NR | NR | NR | NR | NR | No |
| Hindle2016 | NR | Yes | Hypno | NR | NR | NR | NR | Yes | Opioid | No |
| Hopper2015 | NR | Yes | Hypno | NR | NR | NR | NR | NR | NR | No |
| Hoshi2016 | NR | Yes | NR | NR | NR | NR | NR | NR | NR | No |
| Hou2019 | NR | Yes | Hypno | NR | NR | NR | NR | NR | NR | No |
| Howard2015 | NR | NR | NR | NR | NR | NR | NR | NR | NR | No |
| Hsieh2018 | NR | Yes | NR | NR | NR | NR | NR | NR | NR | No |
| Husby2016 | NR | Yes | Disso | Yes | NSAID | NR | NR | Yes | NSAID | No |
| Jakobsen2017 | NR | Yes | NR | NR | NR | NR | NR | NR | NR | No |
| Jia2018 | NR | Yes | Disso | NR | NR | NR | NR | Yes | Opioid + NSAID + local | No |
| Jin2020 | NR | Yes | Hypno | NR | NR | NR | NR | NR | NR | No |
| Kazemi2017 | NR | Yes | Hypno | NR | NR | NR | NR | Yes | NSAID | No |
| Kazemi2015 | NR | Yes | Hypno | Yes | Other | NR | NR | Yes | NSAID | No |
| Keller2019 | NR | NR | NR | NR | NR | NR | NR | Yes | Opioid + NSAID + local | No |
| Kim2018 | NR | Yes | Disso | NR | NR | NR | NR | NR | NR | No |
| Kim2015 | NR | NR | NR | NR | NR | NR | NR | NR | NR | No |
| Kim2020 | NR | Yes | Disso | NR | NR | NR | NR | Yes | Opioid | No |
| Kitamura2016 | NR | Yes | Disso | NR | NR | NR | NR | NR | NR | No |
| Kok2019 | NR | Yes | Hypno | Yes | Opioid + NSAID + local | NR | NR | Yes | Opioid + NSAID + local | No |
| Kon2015 | NR | Yes | NR | NR | NR | NR | NR | Yes | NR | No |
| Kon2020 | NR | Yes | NR | NR | NR | NR | NR | Yes | NSAID | No |
| Kondo2019 | NR | Yes | Inhal | NR | NR | NR | NR | NR | NR | No |
| Korthagen2019 | Yes | Yes | Disso | Yes | Opioid + NSAID + local | Yes | Other | Yes | Opioid + NSAID + local | Yes |
| Lan2016 | NR | Yes | Disso | NR | NR | NR | NR | NR | NR | No |
| Levingstone2016 | Yes | Yes | Hypno | Yes | Opioid + NSAID + local | NR | NR | Yes | Opioid + NSAID + local | No |
| Li2016 | NR | Yes | Hypno | NR | NR | NR | NR | NR | NR | No |
| Li2018 | NR | Yes | Hypno | NR | NR | NR | NR | Yes | Opioid | No |
| Lin2017 | NR | Yes | Disso | NR | NR | NR | NR | NR | NR | No |
| Lin2020 | Yes | Yes | Inhal | Yes | NSAID | NR | NR | Yes | NSAID | No |
| Ling2020 | NR | NR | NR | NRR | NR | NR | NR | NR | NR | No |
| Little2016 | Yes | Yes | Hypno | Yes | Local | Yes | Opioid | Yes | Opioid | Yes |
| Lv2015 | NR | Yes | Hypno | NR | NR | NR | NR | NR | NR | No |
| Lydon2019 | NR | Yes | Hypno | Yes | NSAID | NR | NR | NR | NR | No |
| Madry2020 | NR | NR | NR | NR | NR | NR | NR | NR | NR | No |
| Mancini2020 | Yes | Yes | Disso | Yes | Opioid | Yes | Local | Yes | NSAID | Yes |
| Mancini2017 | NR | Yes | Disso | Yes | Opioid | NR | NR | Yes | Opioid + NSAID + local | No |
| Maninchedda2015 | NR | Yes | NR | NR | NR | Yes | NSAID | Yes | NSAID | No |
| Manunta2016 | NR | Yes | Sedative | Yes | Local | NR | NR | Yes | NSAID | No |
| Marteau2015 | NR | NR | NR | NR | NR | NR | NR | NR | NR | No |
| Martinez-Carranza2016 | NR | Yes | Disso | NR | NR | NR | NR | Yes | NSAID | No |
| Martinez-Carranza2019 | NR | NR | NR | NR | NR | NR | NR | NR | NR | No |
| McCarrel2017 | NR | Yes | NR | NR | NR | Yes | Local | NR | NR | No |
| McCarty2016 | NR | Yes | Hypno | Yes | Opioid | NR | NR | Yes | Opioid | No |
| Meppelink2016 | NR | NR | NR | NR | NR | NR | NR | NR | NR | No |
| Mickevicius2020 | NR | NR | NR | NR | NR | NR | NR | NR | NR | No |
| Miki2016 | NR | Yes | Disso | NR | NR | NR | NR | NR | NR | No |
| Mohan2015 | NR | Yes | Disso | NR | NR | NR | NR | NR | NR | No |
| Moller2020 | Yes | Yes | Disso | Yes | Opioid | NR | NR | Yes | NSAID | No |
| Moore2018 | NR | Yes | NR | NR | NR | NR | NR | NR | NR | No |
| Mrosek2016 | NR | Yes | NR | NR | NR | NR | NR | Yes | Opioid + NSAID + local | No |
| Muhonen2016 | NR | Yes | Hypno | Yes | Opioid + NSAID + local | NR | NR | NR | NR | No |
| Mumme2016 | NR | Yes | NR | NR | NR | NR | NR | NR | NR | No |
| Murata2018 | NR | Yes | Inhal | Yes | NR | NR | NR | NR | NR | No |
| Murata2015 | NR | Yes | NR | NR | NR | NR | NR | NR | NR | No |
| Nelson2019 | NR | Yes | NR | NR | NR | NR | NR | NR | NR | No |
| Nelson2020 | NR | Yes | Disso | NR | NR | NR | NR | NR | NR | No |
| Nie2020 | NR | Yes | NR | NR | NR | NR | NR | NR | NR | No |
| Niemela2019 | Yes | Yes | NR | NR | NR | NR | NR | NR | NR | No |
| Nixon2017 | NR | NR | NR | NR | NR | NR | NR | NR | NR | No |
| Novak2016 | NR | NR | NR | NR | NR | NR | NR | NR | NR | No |
| Olesen2020 | NR | Yes | Hypno | Yes | Other | NR | NR | Yes | Opioid | No |
| Olesen2019 | NR | Yes | Hypno | Yes | Other | NR | NR | NR | NR | No |
| Olive2020 | NR | Yes | Disso | Yes | Local | NR | NR | Yes | NSAID | No |
| Oliver2020 | Yes | Yes | NR | NR | NR | NR | NR | Yes | Opioid | No |
| Onodera2020 | NR | Yes | Disso | NR | NR | NR | NR | NR | NR | No |
| Orth2016 | Yes | Yes | Hypno | NR | NR | NR | NR | Yes | Opioid + NSAID + local | No |
| Orth2020 | NR | Yes | NR | NR | NR | NR | NR | NR | NR | No |
| Ortved2015 | NR | Yes | NR | NR | NR | NR | NR | NR | NR | No |
| Park2015 | NR | Yes | Inhal | NR | NR | NR | NR | NR | NR | No |
| Passino2017 | Yes | Yes | Hypno | Yes | Opioid | Yes | Local | NR | NR | No |
| Patel2020 | NR | Yes | NR | NR | NR | NR | NR | Yes | NR | No |
| Peal2020 | NR | NR | NR | NR | NR | NR | NR | NR | NR | No |
| Peck2015 | NR | Yes | Inhal | NR | NR | NR | NR | NR | NR | No |
| Peretti2017 | NR | Yes | NR | NR | NR | NR | NR | NR | NR | No |
| Perez-Silos2019 | NR | Yes | NR | NR | NR | NR | NR | NR | NR | No |
| Pfeifer2015 | NR | Yes | Inhal | Yes | Opioid | NR | NR | Yes | Opioid + NSAID + local | No |
| Pfeifer2017 | NR | Yes | Disso | NR | NR | Yes | Opioid | Yes | Opioid + NSAID + local | No |
| Pilichi2018 | NR | NR | NR | NR | NR | NR | NR | NR | NR | No |
| Qiao2020 | NR | Yes | Hypno | Yes | Opioid | NR | NR | NR | NR | No |
| Reesink2018 | NR | NR | NR | NR | NR | NR | NR | NR | NR | No |
| Reesink2017 | NR | NR | NR | NR | NR | NR | NR | NR | NR | No |
| Ribitsch2018 | NR | NR | NR | Yes | Opioid | NR | NR | NR | NR | No |
| Roth2020 | NR | NR | NR | NR | NR | NR | NR | NR | NR | No |
| Ruvinov2019 | NR | Yes | NR | NR | NR | NR | NR | NR | NR | No |
| Sabry2018 | NR | Yes | NR | NR | NR | NR | NR | NR | NR | No |
| Salkeld2016 | NR | NR | NR | NR | NR | NR | NR | Yes | Opioid | No |
| Salonius2019 | NR | Yes | NR | Yes | NSAID | NR | NR | NR | NR | No |
| Santos2019 | NR | NR | NR | NR | NR | NR | NR | NR | NR | No |
| Sarin2020 | NR | NR | NR | NR | NR | NR | NR | NR | NR | No |
| Sarin2018 | Yes | NR | NR | NR | NR | NR | NR | NR | NR | No |
| Schagemann2016 | Yes | Yes | Disso | Yes | Local | NR | NR | Yes | NSAID | No |
| Schell2019 | NR | Yes | NR | Yes | Opioid | NR | NR | NR | NR | No |
| Schinhan2020 | NR | NR | NR | NR | NR | NR | NR | NR | NR | No |
| Schone2016 | NR | Yes | NR | NR | NR | NR | NR | NR | NR | No |
| Schwarz2019 | Yes | Yes | Hypno | Yes | Opioid + NSAID + local | NR | NR | Yes | Opioid + NSAID + local | No |
| Seo2015 | NR | Yes | Hypno | NR | NR | NR | NR | Yes | NSAID | No |
| Sheu2017 | NR | Yes | Disso | Yes | Opioid + NSAID + local | NR | NR | NR | NR | No |
| Silva2018 | NR | Yes | Hypno | Yes | Opioid | NR | NR | Yes | Opioid | No |
| Smith2016 | Yes | Yes | NR | Yes | NSAID | NR | NR | Yes | NSAID | No |
| Sobol2017 | NR | NR | NR | NR | NR | NR | NR | NR | NR | No |
| Sosio2015 | NR | Yes | Inhal | Yes | NSAID | NR | NR | Yes | NSAID | No |
| Stannard2015 | NR | Yes | Hypno | Yes | Opioid | NR | NR | Yes | Opioid | No |
| Stefani2020 | NR | Yes | NR | Yes | Opioid | NR | NR | Yes | Opioid | No |
| Stefani2020 | NR | Yes | NR | Yes | Opioid | NR | NR | Yes | Opioid | No |
| Stewart2019 | NR | NR | NR | NR | NR | NR | NR | NR | NR | No |
| Sun2016 | NR | Yes | NR | NR | NR | NR | NR | NR | NR | No |
| Sun2020 | NR | Yes | Hypno | NR | NR | NR | NR | NR | NR | No |
| Szivek2017 | NR | NR | NR | NR | NR | NR | NR | NR | NR | No |
| Tang2019 | NR | Yes | Disso | NR | NR | NR | NR | NR | NR | No |
| Theruvath2019 | NR | Yes | Inhal | NR | NR | NR | NR | NR | NR | No |
| Tomaszewski2019 | Yes | Yes | Hypno | Yes | Opioid | NR | NR | NR | NR | No |
| Toth2015 | NR | Yes | Disso | NR | NR | NR | NR | NR | NR | No |
| Tothova2019 | NR | Yes | Disso | Yes | Opioid | NR | NR | Yes | NSAID | No |
| Tothova2019 | NR | Yes | Disso | Yes | Opioid | NR | NR | Yes | NSAID | No |
| Tuska2016 | NR | NR | NR | Yes | NSAID | NR | NR | Yes | NSAID | No |
| Uklejewski2016 | NR | Yes | Disso | Yes | NSAID | NR | NR | NR | NR | No |
| Uto2018 | NR | Yes | NR | NR | NR | NR | NR | NR | NR | No |
| Vahedi2020 | NR | Yes | Sedative | Yes | Other | NR | NR | NR | NR | No |
| Vahedi2019 | NR | Yes | Sedative | Yes | Other | NR | NR | NR | NR | No |
| Vikingsson2015 | NR | Yes | NR | NR | NR | NR | NR | NR | NR | No |
| Vindas Bolanos2017 | NR | Yes | Disso | NR | NR | NR | NR | Yes | NSAID | No |
| Wang2019 | NR | Yes | Hypno | NR | NR | NR | NR | NR | NR | No |
| Wang2017 | NR | Yes | Disso | NR | NR | NR | NR | NR | NR | No |
| Wei2019 | NR | Yes | Hypno | NR | NR | NR | NR | NR | NR | No |
| Wiegant2015 | NR | Yes | NR | Yes | Opioid + NSAID + local | NR | NR | Yes | NSAID | No |
| Wiegant2016 | NR | Yes | NR | NR | NR | NR | NR | Yes | NR | No |
| Wong2020 | NR | Yes | Sedative | NR | NR | NR | NR | NR | NR | No |
| Wu2019 | NR | Yes | NR | NR | NR | NR | NR | NR | NR | No |
| Wu2018 | NR | Yes | Disso | NR | NR | NR | NR | NR | NR | No |
| Wu2019 | NR | Yes | NR | NR | NR | NR | NR | NR | NR | No |
| Xiao2019 | Yes | Yes | Hypno | NR | NR | NR | NR | NR | NR | No |
| Xin2020 | NR | NR | NR | NR | NR | NR | NR | NR | NR | No |
| Xu2020 | NR | Yes | Hypno | NR | NR | NR | NR | NR | NR | No |
| Xu2020 | NR | NR | NR | NR | NR | NR | NR | NR | NR | No |
| Xue2018 | NR | NR | NR | NR | NR | NR | NR | NR | NR | No |
| Yamasaki2018 | NR | Yes | Disso | Yes | Opioid | NR | NR | Yes | NSAID | No |
| Yamasaki2019 | NR | Yes | Disso | NR | NR | NR | NR | NR | NR | No |
| Yamasaki2015 | NR | Yes | Disso | NR | NR | NR | NR | NR | NR | No |
| Yan2019 | NR | Yes | Hypno | NR | NR | NR | NR | NR | NR | No |
| Yan2017 | Yes | Yes | Disso | NR | NR | NR | NR | NR | NR | No |
| Yoon2016 | NR | Yes | Inhal | Yes | NSAID | NR | NR | Yes | NSAID | No |
| Yu2020 | Yes | Yes | Hypno | Yes | Local | NR | NR | NR | NR | No |
| Yucekul2017 | NR | NR | NR | NR | NR | NR | NR | NR | NR | No |
| Zedde2016 | NR | Yes | Hypno | Yes | Opioid | NR | NR | NR | NR | No |
| Zedde2017 | Yes | Yes | Hypno | Yes | Opioid | NR | NR | NR | NR | No |
| Zhai2018 | NR | NR | NR | NR | NR | NR | NR | NR | NR | No |
| Zhang2020 | NR | Yes | NR | NR | NR | NR | NR | Yes | NR | No |
| Zhang2018 | NR | Yes | Hypno | NR | NR | NR | NR | NR | NR | No |
| Zhang2020 | NR | Yes | Disso | NR | NR | NR | NR | NR | NR | No |
| Zhang2018 | NR | Yes | Disso | NR | NR | NR | NR | NR | NR | No |
| Zhang2020 | NR | Yes | Hypno | Yes | Other | Yes | Opioid | NR | NR | No |
| Zhang2017 | NR | NR | NR | NR | NR | NR | NR | NR | NR | No |
| Zhang2019 | NR | Yes | NR | NR | NR | NR | NR | NR | NR | No |
| Zhao2018 | NR | Yes | Hypno | NR | NR | NR | NR | NR | NR | No |
| Zhu2017 | NR | Yes | NR | NR | NR | NR | NR | NR | NR | No |
| Zorzi2015 | NR | Yes | Hypno | Yes | Opioid | NR | NR | NR | NR | No |
| Zuo2016 | NR | Yes | Disso | NR | NR | NR | NR | NR | NR | No |
| Monit = monitoring, Anaes = Anaesthesia, Analg = Analgesia, Pre-op = Pre-operative, In=op = Intra=operative, Post=op = Post-operative, NR = Not Reported, Disso = Dissociative (e.g. Ketamine, Tiletamine), Inhal = Inhalant (e.g. Isofluorane, Sevofluorane), Hypno = Hypnotic (e.g. Alfaxalone, Propofol, Barbiturates), Sedative = Sedative only, NSAID = Non-Steroidal Anti-Inflammatory Drug, Local = Local analgesic | | | | | | | | | | |

Supplementary File 3: Section on surgical procedures in Item 9 of the ARRIVE guidelines^16^


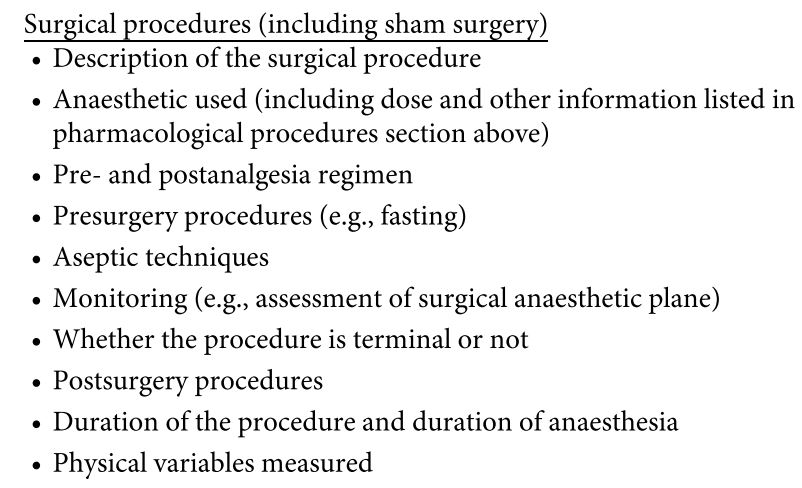


Supplementary file 4: Correlation between A) reporting of pre-,intra- or postoperative analgesia and year of publication and B) reporting of anaesthesia and year of publication.
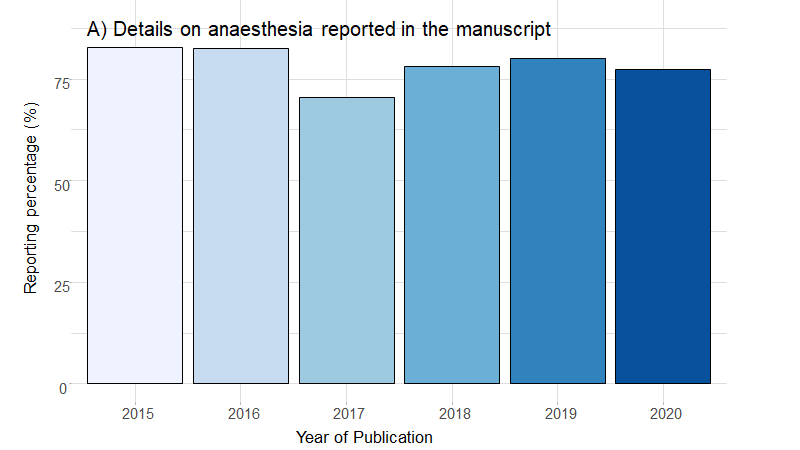


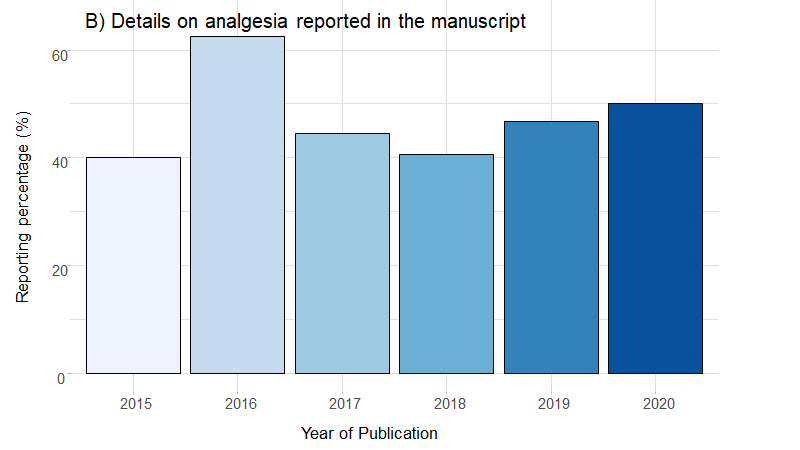

Supplement: Multimedia component 1 [file mmc1.docx]
